# Childhood socioeconomic background and elevated mortality among the young adult second generation in Sweden: a population-based cohort study

**DOI:** 10.1136/bmjph-2023-000643

**Published:** 2024-05-27

**Authors:** Matthew Wallace, Eleonora Mussino, Siddartha Aradhya, Lisa Harber-Aschan, Ben Wilson

**Affiliations:** 1 Stockholm University, Stockholm, Stockholm County, Sweden

**Keywords:** Sociodemographic Factors, Public Health, Epidemiology, Humans

## Abstract

**Introduction:**

The native-born children of migrants represent one of the fastest-growing and most diverse young populations in the world today. A growing body of research highlights an elevated young adult mortality risk in this ‘second generation’ (G2) relative to the majority population at the same ages. Previous studies have tried to understand this increased risk by examining its association with inequality in the adult socioeconomic background (SEB) of the G2. Here, we instead analyse the association of second-generation status with childhood SEB.

**Methods:**

We use administrative register data from Sweden to fit multistate, competing-risk, flexible parametric survival models on a data set of 13 404 deaths in 2.35 million young adults. We examine mortality from all causes and specific causes of death at the generational level and by parental region of birth, both before and after having adjusted for childhood SEB.

**Results:**

The G2 have higher all-cause mortality hazard rates (HR=1.29 (95% CIs=1.23 to 1.34)) than the majority population before adjusting for childhood SEB. Following adjustment, the size of the hazard rate is smaller, but remains higher than the majority population (aHR=1.16 (95% CIs=1.11 to 1.21)). The G2 additionally experience persistent and higher hazard rates of mortality from suicide (aHR=1.29 (95% CIs=1.20 to 1.39)), substance misuse (aHR=1.41 (95% CIs=1.26 to 1.58)) and assault (aHR=2.54 (95% CIs=2.02 to 3.20)). By parental origins, similar patterns to those described are documented among G2 that have at least one parent born in Finland, the other Nordic countries, former Yugoslavia, the rest of Europe, sub-Saharan Africa, Northern Africa, and Iran and Iraq. However, higher all-cause (aHR=1.42 (95% CIs=1.33 to 1.51)) and external-cause hazard rates of mortality (aHR=1.59 (95% CIs=1.48 to 1.72)) only persist among G2 with parent(s) born in Finland.

**Conclusions:**

G2 with various parental origins have higher mortality rates than the majority population do, and this difference is partly explained by their childhood SEB.

**WHAT IS ALREADY KNOWN ON THIS TOPIC**

- In general populations, lower childhood socioeconomic background (SEB) is associated with an elevated young adult mortality risk, independent of adult SEB. The native-born children of immigrants (the ‘second generation’ (G2)) have increased all-cause and external-cause mortality risks compared with the majority population born in the host country. This contrasts with the lower young adult mortality risks of first-generation migrants from a diverse range of origin countries living in many host countries. Considering that the childhood SEB of the first and G2s in childhood typically diverge (ie, SEB advantage among the first generation during their formative years living in the country of birth and SEB disadvantage among the G2 during their formative years in their parent’s host country), its role in the increased mortality risks of the G2 represents an as-yet-unexplored area of interest.

**WHAT THIS STUDY ADDS**

- G2 with various parental origins have higher mortality rates than the majority population. This difference is partly explained by their childhood SEB at the generational level and fully explained for the G2 with at least one parent born in former Yugoslavia, the rest of Europe, sub-Saharan Africa, Northern Africa, and Iran and Iraq.

**HOW THIS STUDY MIGHT AFFECT RESEARCH, PRACTICE OR POLICY**

- Public health and social policies that seek to improve the mortality situation of an adult G2 that is growing in many high-income countries must adopt an intergenerational approach to address these increased mortality risks. Addressing inequalities between the adult SEB of first-generation migrants and the majority population could help to reduce the young adult mortality risks of second-generation children.

## Introduction

 The second generation (G2; children of migrants) are most commonly defined as people born in a country to at least one foreign-born parent.^1^ This group represents one of the fastest-growing and most diverse young populations in migrant-receiving countries in the world today, due to the establishment and continuation of decades of international migration.^2^ In the European Union (EU) in 2021, 7% of the population aged 15–74 years were G2. These proportions were considerably higher in late adolescence and young adulthood (15–29 years; 11%).^3^ Sweden, the context of this study, has some of the highest proportions of G2 in all of the EU at 12% (15–74 years) and 19% (15–29 years).^3^ A recent review of mortality among the G2 in Europe describes an elevated risk of death in young to middle adulthood (between ages 15 and 64 years) compared with people born in a country to two parents born in that same country (a group henceforth referred to as the majority population).^4^ G2 people—especially men—with parent(s) born outside of Europe, particularly the Middle East, Northern Africa and sub-Saharan Africa, have elevated mortality risks between these ages compared with the majority population.^4^

Previous research has investigated this higher adult mortality risk of the G2 by focusing on its association with inequalities in their *adult* socioeconomic background (SEB)—particularly educational and labour market outcomes.^5^,^9^ These studies show that adult socioeconomic inequality among the G2 often explains a substantial part, but not all, of their higher mortality risk.^4^ However, this only explores a part of the lives of the G2. Less research has focused on the association of the higher adult mortality risks of the G2 with their *childhood* SEB,^10^ a critical, formative part of the life course. The ‘long arm’ of childhood SEB affects mortality well into adult life, independent of adult SEB.^11^,^14^ Migrants (the parents of the G2) typically experience downward social mobility after arriving in the host country. They are exposed to varying degrees (according to their country of birth) of socioeconomic inequality relative to the majority population.^15^,^17^ It is *these* conditions that directly inform the childhood SEB of the G2.

Additionally, there is evidence of *upward* intergenerational mobility among the G2 in Europe, particularly among children of migrants who have lower SEB origins.^15^,^17^ This means that parental SEB (and thus childhood SEB) might differ substantially from the adult SEB of those upwardly mobile G2.^11^ Thus, only focusing on associations between adult SEB and the higher mortality risks of the G2 might fail to capture the childhood socioeconomic disadvantage that the G2 were exposed to. Childhood SEB has an influence on mortality beyond its direct influence on adult SEB. This indicates that childhood SEB and adult SEB could have independent impacts on mortality among the G2 in addition to an impact through their effect on one another.^12^,^14^ We thus conceptualise that childhood SEB (captured by parental SEB) affects the mortality of the G2, both through its effect on adult SEB and through other mechanisms. This is in addition to any independent effects of adult SEB—which are not the focus of this analysis. We follow prior research in arguing that we expect these interrelationships to be contingent on a number of factors, including demographic factors such as sex, birth cohort and the parental country of birth, as well as the context in which our study takes place.

The aim of this article is to investigate the association of childhood SEB with variation in the young adult mortality risks of the G2 compared with the majority population in Sweden, with a focus on specific parental origins and causes of death.

### Data & Methods

#### Data

We used the collection of Swedish register data *Ageing Well* at Stockholm University. The collection covers longitudinal, individual-level data from a range of administrative sources. We used the total population register, migration register, multigenerational register, cause-of-death register and the *Longitudinal Integrated Database for Health Insurance and Labour Market Studies* (LISA). Information was linked between the same people across the different register sources using a unique individual identifier. *Ageing Well* was generated and pseudo-anonymised for research purposes.

To be included in the study, subjects had to be (a) born in Sweden, (b) turn age 16 years between 1 January 1992 and 31 December 2016 and (c) have at least one living parent on turning 16 years old (to derive the parental information). A delayed entry design was imposed; entry into the study was conditional on survival to 16 years old.

The outcomes were all-cause mortality and mortality from specific causes. The age at death (derived from the exact date of death in the *cause-of-death register*) was used to identify whether someone had died. The cause of death was derived from the underlying cause-of-death variable in the *cause-of-death register* and categorised into two variables with differing detail levels. Variable one coded the cause of death into natural and external causes. Variable two coded the cause of death into 12 cause groups: cancer, circulatory diseases, respiratory diseases, endocrine, nutritional and metabolic diseases, diseases of the nervous system, other diseases and medical conditions, suicides, substance misuse (including alcohol), traffic accidents, other accidents and injuries, assault and unknown causes of mortality. The causes were coded using a combination of International Classification of Diseases 9th Revision (ICD-9) (1992–1996) and ICD-10 (1997—) codes. Online supplemental table S2 shows exactly how we categorised the causes of death.

Exposure was G2 status. People were classed as G2 if they were born in Sweden to *at least* one parent born abroad. G2 status was generated by linking parents to children (via the *multigenerational register* and the matching of parental and child identifications) and using information on individual and parental country of birth (derived from the *total population register*). The reference population in the models was always the majority population. We examined the association between childhood SEB and mortality at the generational level (ie, all G2 combined) and according to more granular parental origins. Specifically, those with at least one parent born in Finland, the other Nordic countries, other (non-Nordic) EU/European Economic Area (EEA) countries, former Yugoslavia, the rest of Europe, South America, sub-Saharan Africa, Northern Africa, Iran and Iraq, other Middle East and Northern Africa (MENA) and Asia.

Predictor variables included sex, birth cohort, family and living situation, highest level of parental education, parental disposable income and parental unemployment status. Family and living situation and the highest level of parental education were measured in the year the child turned 16 years old. Parental disposable income and unemployment status were measured in a 3-year period in which the child was 14, 15 and 16 years of age to counteract some of the volatility linked with single-year measures of these two predictors.

Sex was derived from the *total population register* and grouped into ‘male’ and ‘female’. Birth cohort was from the date of birth in the same register for years 1976 to 2000. Family situation was derived from *LISA* and grouped into ‘living at home with married parents’, ‘living at home with cohabiting parents’, ‘living at home with a single parent’ and ‘lives alone’.

The highest level of parental education (in *LISA*) was coded according to the International Standard Classification of Education into ‘primary’, ‘secondary’ and ‘post-secondary’ levels.

Parental disposable income quintile (from *LISA*) was based on a variable that records disposable income for a calendar year. To begin with, we estimated the average of both parents’ annual disposable incomes for each of the 3 years in which the child was 14, 15 and 16 years old. For each year, we then ranked the averages within centiles, generating a place in a distribution ranging from 0 to 100. The average of the centiles across the 3 years was taken and grouped into ‘lowest’, ‘lower’, ‘medium’, ‘higher’ and ‘highest’ quintiles.

Parental unemployment status (from *LISA*) was based on a variable that records the number of days of unemployment within a calendar year. For a given year, if a parent recorded 90 days or more of unemployment, they were coded as ‘unemployed’. The 90-day cut-off was adopted from a study examining unemployment persistence among the G2 in Sweden, which showed a good degree of consistency between this cut-off and official unemployment statistics from the Swedish Labour Market Survey.^18^ The total number of years unemployed in the 3-year period before the child turns age 16 years was then summed to a value between 0 (ie, 3 years in which a parent records *less than* 90 days of unemployment) and 3 (ie, 3 years in which a parent has *equal to or more than* 90 days of unemployment). An average of the values of the two parents was then taken and organised into unemployed for ‘0 years’, ‘1 year’, ‘2 years’ and ‘3 years’.

We note that there were 69 225 (or 2.86%) cases where a parent died before the child turned 16 years old. In these cases, we only considered the living parent’s education level and living parent’s unaveraged values for disposable income and unemployment status.

#### Methods

We implemented a multistate competing risk approach following the logic outlined in Putter *et al*.^19^ We fitted flexible parametric survival models, assuming a proportional hazard approach using ‘stpm2’ in Stata 18.^20 21^ The HRs were reported alongside 95% CI. In light of several of the well-known shortcomings associated with hazard ratios, several regression-standardised (ie, confounder-adjusted) measures of risk were also reported in online supplemental materials.

People joined the risk set on turning 16 years old during the period 1992–2016. They exited the risk set if they died, emigrated or were alive and residing in Sweden on 31 December 2016. The oldest age reached was 40 years old. Whether or not someone had emigrated was determined from registered emigration in the *migration register* and a residence indicator from the total population register updated at the end of each calendar year.

We conducted a complete case analysis—an analysis that includes only individuals for which there is no missing information in the exposure or any of the predictor variables. From a potentially eligible starting population of 2 368 459 individuals, 2 345 833 individuals (~99%) were retained for our final statistical analysis. 22 626 individuals were removed due to missing data in their family situation (17 763; 0.7%), parental educational level (1732; 0.1%), parental disposable income (3959; 0.2%) and parental unemployment status (3995; 0.2%). Note that these numbers do not sum to 22 626 due to an overlap in missing information across the variables for the same people in the data set.

### Analytical strategy

**Figure 1:**
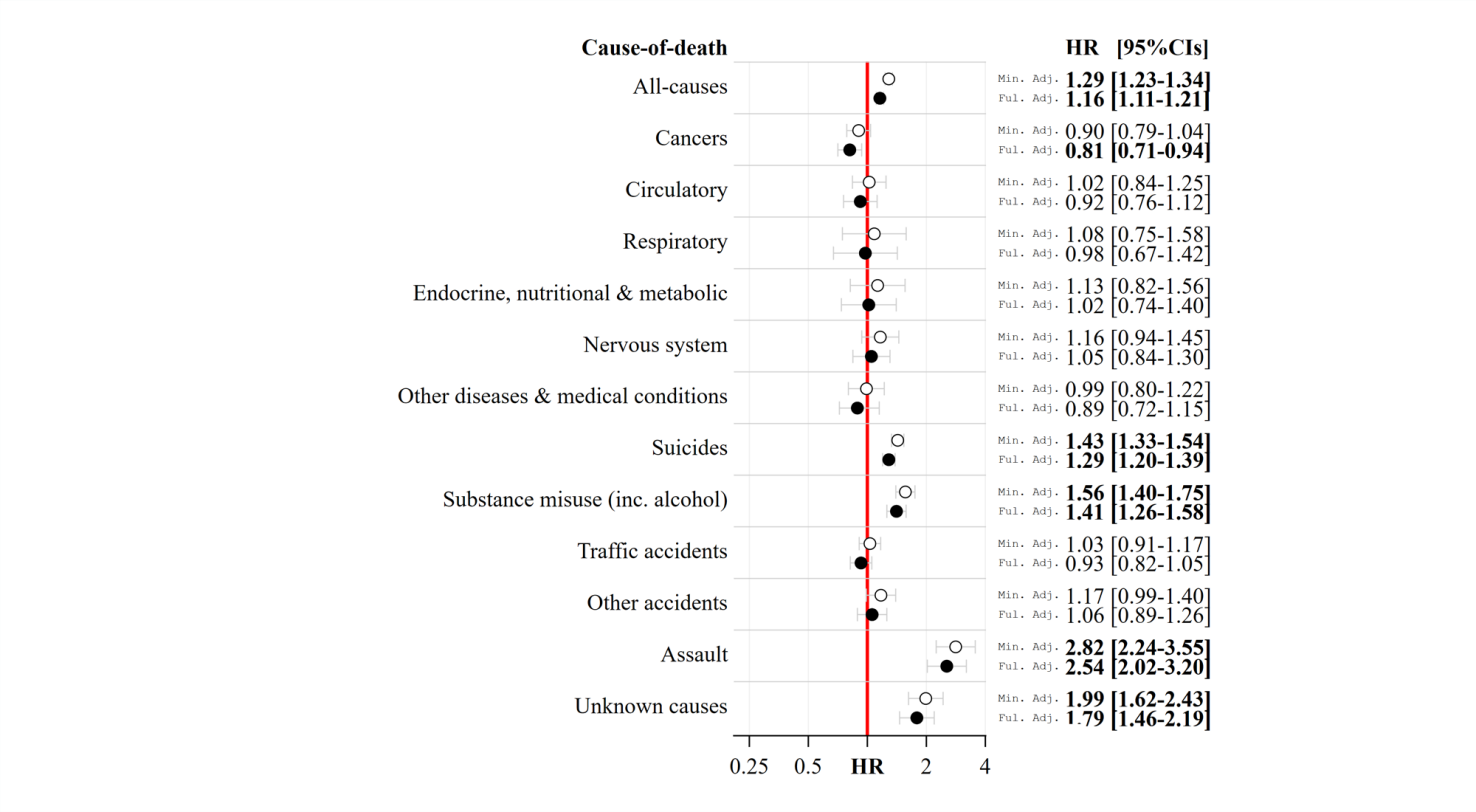
HRs of all-cause mortality and mortality from specific causes of death among the second generation aged 16–40 years in Sweden, 1992–2016, before and after adjusting for childhood socioeconomic background. Notes: Estimates in bold have CIs that do not overlap with 1. The red line represents the majority population reference category. ‘Min. Adj.’ refers to the minimally adjusted model, and ‘Ful. Adj.’ refers to the fully adjusted model. ‘HR’ refers to hazard ratio. Source: author calculations based on Swedish register data Ageing Well.

**Figure 2:**
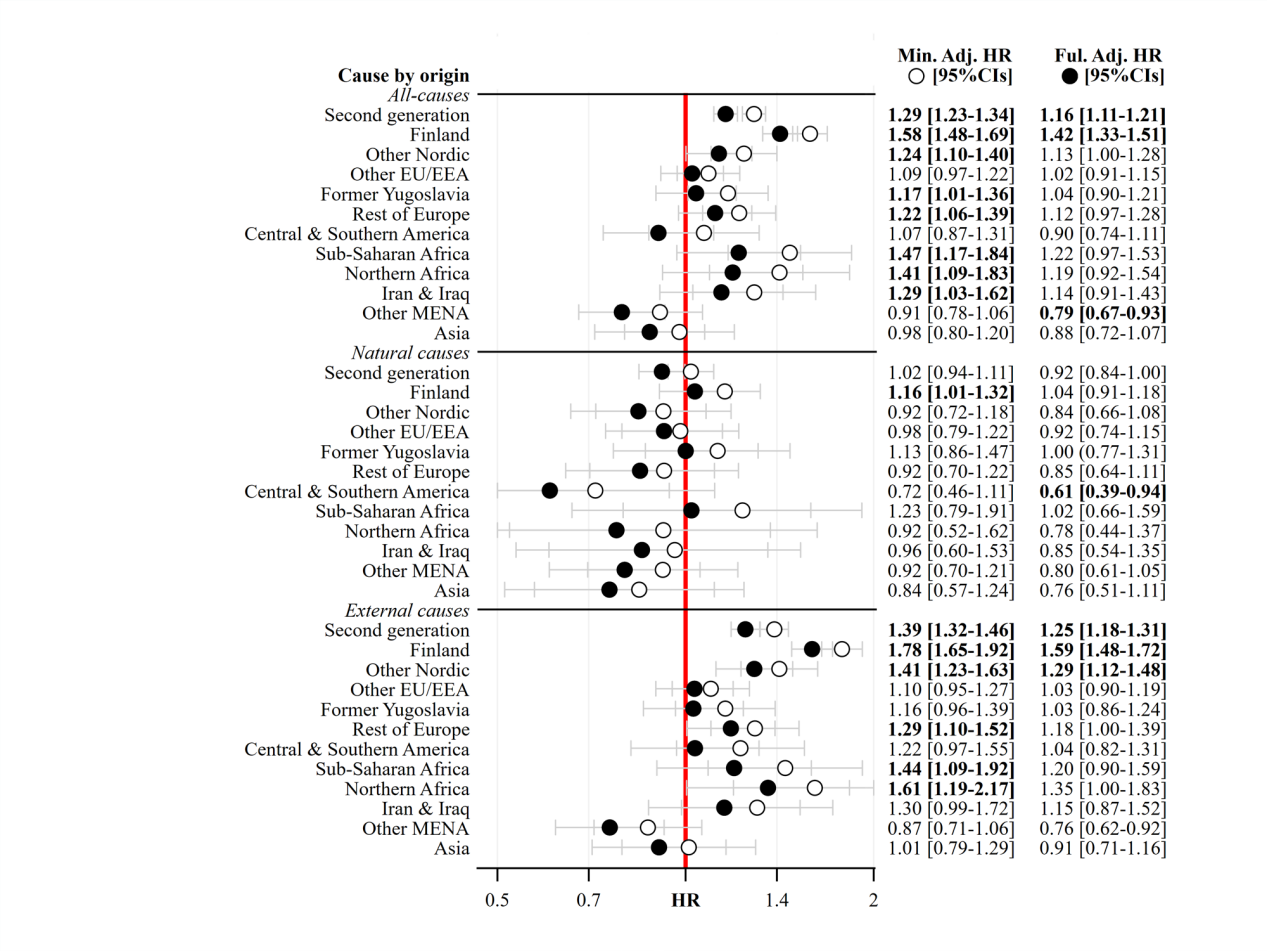
HRs of all-cause, natural and external mortality among the second generation aged 16–40 years in Sweden, 1992–2016, before and after adjusting for childhood socioeconomic background. Notes: Estimates in bold have CIs that do not overlap with 1. The red line represents the majority population reference category. ‘Min. Adj.’ refers to the minimally adjusted model, and ‘Ful. Adj.’ refers to the fully adjusted model. ‘HR’ refers to hazard ratio. Source: author calculations based on Swedish register data Ageing Well.

We fitted two models, a ‘minimally adjusted’ model and a ‘fully adjusted’ model. The minimally adjusted model controlled for birth cohort (in single years, with 1976 as the reference group in the models), sex (with female as the reference group) and G2 status (with the majority population as the reference group). The fully adjusted model controlled for birth cohort, sex and G2 status and additionally controlled for the highest level of parental education (with tertiary level of education as the reference group), parental disposable income (with the highest income quintile as the reference group) and years of parental unemployment (with zero years of unemployment as the reference group). Figure 1 presents the minimally adjusted and fully adjusted HRs of all-cause and cause-specific mortality at the generational level using the 12-category cause-of-death variable. Figure 2 displays the minimally adjusted and fully adjusted HRs of all-cause and cause-specific mortality for the specific parental birth regions using the three category cause-of-death variable. When describing the results, we use ‘HR’ to refer to the minimally adjusted model and ‘aHR’ to refer to the fully adjusted model.

We conducted a large number of additional analyses to supplement and contextualise our main results. Online supplemental table S1 offers descriptive information about the sample (population sizes, time at risk, crude and age-standardised mortality rates) for the parental origin variable by natural and external causes of death. Online supplemental table S2 shows similar information to online supplemental table S1 but at the generational level and for the 12-category cause-of-death variable. Online supplemental table S3 shows how the distribution of the predictors varies between the majority population and the G2. Online supplemental table S4 reports the HRs of the predictor variables from the fully adjusted, all-cause mortality model. Online supplemental table S5 investigates sex differences in the HRs at the generational level. Online supplemental table S6 reports results for a more restrictive definition of the G2, limiting exposure only to people born in Sweden to two parents born abroad. For the regression sensitivity analyses, HRs for online supplemental tables S5 and S6 have also been investigated in one common model (ie, via the use of interaction terms). This did not change the value of the estimates or confidence limits. Online supplemental figure S1 reports annual entries into the population at risk broken down by parental region of birth so as to understand more how the parental origin composition of the G2 population changes over entry year. Online supplemental figures S2–S5 display a series of regression-standardised survival probabilities, failure probabilities, mortality rates and cumulative incidence functions (CIFs) from the generational-level models to allow us to interpret our main findings in terms of absolute risk.

## Results

Online supplemental table S1 shows descriptive information on the population sizes, time at risk (in years), deaths and crude and age-standardised death rates—standardised to the age profile of the majority population of Sweden. The average crude mortality rates of the majority population are 43 people per 100 000 (all cause), 13 people per 100 000 (natural) and 29 people per 100 000 (external). There are clear differences between the mortality rates of the G2 and the majority population. At the generation level, the age-standardised rates for the G2 are 56 people per 100 000 (all-cause mortality), 14 people per 100 000 (natural-cause mortality) and 39 people per 100 000 (external-cause mortality). By specific parental regions of birth, many groups have higher all-cause and external mortality rates when compared with the majority population. In particular, G2 with parent(s) born in Finland (all cause 70 per 100 000; external 53 per 100 000), sub-Saharan Africa (all cause 65 per 100 000; external 41 per 100 000) and Northern Africa (all cause 64 per 100 000; external causes 47 per 100 000). Online supplemental table S2 provides the same information at the generational level for the 12-category cause-of-death variable.

Online supplemental table S3 gives the distribution of childhood SEB at the generational level and by parental birth country. It reveals a generalised pattern of disadvantage for all G2 compared with the majority population. Greater proportions of G2 (a) live with a single parent, (b) have parents with primary education only, (c) have parents that occupy the lower and lowest disposable income quintiles and (d) have parents who had at least some unemployment. These general patterns are most pronounced among the G2 with parent(s) born in non-Western countries (Central and Southern America to Asia). G2 with parent(s) born in other EU/EEA countries have the most comparable SEB with the majority population of Sweden. Limited exceptions are the higher proportions of postsecondary education among G2 with parent(s) born in the rest of Europe and Iran and Iraq.

Figure 1 shows the minimally adjusted and fully adjusted all-cause and cause-specific HRs (for 12 causes of death) for the G2 at the generational level. The HRs represent the ratio of the hazard rate for all-cause mortality, or a given cause of death, among the G2 versus the respective hazard rate of the majority population before and after adjusting for childhood SEB. The hazard rate of all-cause mortality in the G2 is higher than the majority population in the minimally adjusted survival model (HR=1.29 (95% CIs=1.23 to 1.34)). It remains higher in the fully adjusted model, even if the HR is closer to one (aHR=1.16 (95% CIs=1.11 to 1.21)). For disease-related causes of death (cancer, circulatory, respiratory, endocrine, nutritional and metabolic, diseases of the nervous system and other diseases and medical conditions), the cause-specific hazard rates of the G2 and majority population are similar in both the minimally adjusted and fully adjusted models. Cancer provides an exception as the G2 hazard rate is lower (aHR=0.81 (95% CIs=0.71 to 0.94)). For the external cause-of-death groups, the hazard rate of suicide mortality is higher than the majority population in the minimally adjusted model; it remains higher in the fully adjusted models (aHR=1.29 (95% CIs=1.20 to 1.39)). The same pattern also applies to mortality from substance misuse (aHR=1.41 (95% CIs=1.26 to 1.58)), assault (aHR=2.54 (95% CIs=2.02 to 3.20)) and for unknown causes of death (aHR=1.79 (95% CIs=1.46 to 2.19)).

Figure 2 shows the minimally adjusted and fully adjusted all-cause and cause-specific HRs (for two broad cause-of-death groupings: natural and external causes of death) for the G2 by specific parental region of birth. In the minimally adjusted model, hazard rates of all-cause mortality are higher among G2 who have parent(s) who were born in Finland (HR=1.58 (95% CIs=1.48 to 1.69)), the other Nordic countries (HR=1.24 (95% CIs=1.10 to 1.40)), former Yugoslavia (HR=1.17 (95% CIs=1.01 to 1.36)), the rest of Europe (HR=1.22 (95% CIs=1.06 to 1.39)), sub-Saharan Africa (HR=1.47 (95% CIs=1.17 to 1.84)), Northern Africa (HR=1.41 (95% CIs=1.09 to 1.83)) and Iran and Iraq (HR=1.29 (95% CIs=1.03 to 1.62)) than the majority population reference category. In the fully adjusted models, only the hazard rate of all-cause mortality among G2 with parent(s) born in Finland (aHR=1.42 (95% CIs=1.33 to 1.51)) remains higher than the majority population. For those with at least one parent born in other Middle Eastern and Northern African countries, the hazard rate of all-cause mortality is actually lower than the majority population in the fully adjusted model (aHR=0.79 (95% CIs=0.67 to 0.93)).

For natural causes, the hazard rates of mortality among the G2 groups are similar to the majority population in figure 2. Nevertheless, the natural cause mortality hazard rate *is* higher among G2 with parent(s) born in Finland (HR=1.16 (95% CIs=1.01 to 1.32)) in the minimally adjusted model. This is not the case in the fully adjusted model (aHR=1.04 (95% CIs=0.91 to 1.18)). For external causes, G2 with parent(s) born in Finland (HR=1.78 (95% CIs=1.65 to 1.92)), the other Nordic countries (HR=1.41 (95% CIs=1.23 to 1.63)), the rest of Europe (HR=1.29 (95% CIs=1.10 to 1.52)), sub-Saharan Africa (HR=1.44 (95% CIs=1.09 to 1.92)) and Northern Africa (HR=1.61 (95% CIs=1.19 to 2.17)) have higher hazard rates than the majority population in the minimally adjusted model. Only the external-cause mortality hazard rates of G2 with parent(s) who were born in Finland (aHR=1.59 (95% CIs=1.48 to 1.72)) and the other Nordic countries (aHR=1.29 (95% CIs=1.12 to 1.48)) remain higher than the majority population in the fully adjusted models.

We conducted various sensitivity analyses to help interpret the results. Online supplemental figure S1 provides the absolute and relative number of entries into the population risk set (respectively) broken down by the parental region of birth. It shows that the G2 with parents who were born in other parts of Europe (ie, Finland, the other Nordic countries, other EU/EEA countries, former Yugoslavia and the rest of Europe) enter the population risk set *earlier* (and thus belong to older birth cohorts) than those G2 with parents born in non-European countries (who thus belong to the younger birth cohorts included in the analysis).

Online supplemental table S4 shows HRs for family situation, parental education, parental income and parental unemployment from the fully adjusted all-cause mortality survival models. The hazard rate is higher among individuals whose highest level of parental education is secondary (aHR=1.24 (95% CIs=1.20 to 1.29)) or primary (aHR=1.27 (95% CIs=1.13 to 1.41)) compared with those whose highest level of education is tertiary. Hazard rates are higher among individuals whose parents belong to the lower (aHR=1.16 (95% CIs=1.09 to 1.24)) and lowest (aHR=1.35 (95% CIs=1.26 to 1.44)) disposable income quintiles relative to those with parents in the highest disposable income quintile. All of those whose parents experienced at least some unemployment have higher hazard rates than those whose parents have not endured unemployment. The hazard rate is highest among those whose parents had 3 years of unemployment (aHR=1.65 (95% CIs=1.38 to 1.94)).

Online supplemental table S5 gives sex-specific estimates for all-cause and cause-specific mortality at the generational level. The hazard rate of all-cause mortality among G2 men is higher when compared with the majority population of men in the fully adjusted model (aHR=1.14 (95% CIs=1.08 to 1.20)). The same is true among G2 women relative to the majority of women in the fully adjusted model (aHR=1.21 (95% CIs=1.11 to 1.31)). The main difference to report in the specific causes of death is that the hazard rate of assault mortality is only higher among men in the fully adjusted survival model (aHR=3.37 (95% CIs=2.59 to 4.38)).

Online supplemental table S6 gives estimates according to whether both of the parents are foreign born (the ‘G2’) or just one parent born is foreign born (the ‘G2.5’). The all-cause mortality hazard rates among the G2 (aHR=1.16 (95% CIs=1.08 to 1.24)) and the G2.5 (aHR=1.16 (95% CIs=1.10 to 1.22)) are comparable to the all-cause mortality hazard rate of figure 1’s main definition (aHR=1.16 (95% CIs=1.11 to 1.21)). There is consistency between definitions by cause of death. Simultaneously, the assault mortality hazard rate (aHR=4.55 (95% CIs=3.49 to 5.93)) is highly elevated among the G2 when compared with the main definition (aHR=2.54 (95% CIs=2.02 to 3.20)). Moreover, assault mortality is not elevated in the G2.5 relative to the majority population (aHR=1.13 (95% CIs=0.77 to 1.65)).

Online supplemental figures S2–S4 display the regression standardised survival probability (online supplemental figure S2), the failure probability (online supplemental figure S3) and mortality rates per 100 000 person-years (online supplemental figure S4) with 95% CIs. A table of estimates is also provided. The measures were postestimated after the fully adjusted, generational-level, all-cause mortality model. For the survival probability, failure probability and mortality rates, the CIs of the majority population and G2 do not overlap for large parts of the age range. The G2 display a lower survival probability, a higher risk of failure and higher death rates compared with the majority population. The gap in each metric widens over age. For example, the mortality rates show a difference of 1.4 deaths per 100 000 person-years at age 16 years (95% CIs=1.2 to 1.7) that rise to 11 deaths per 100 000 person-years at 40 years (95% CIs=9.7 to 12.5). This analysis also shows precisely how rare mortality is in our sample population. By age 40 years, the risk of all-cause mortality is 1.15% (95% CIs=1.11% to 1.18%) in the majority population and 1.33% (95% CIs=1.27% to 1.39%) in the G2, a difference of 0.18% pt (95% CIs=0.12% to 0.24%).

Online supplemental figure S5 shows regression-standardised CIFs for the 12 causes of death at generational level. In the context of the HRs that we reported in the text earlier from figure 1, here, we report the CIFs for suicide, substance misuse and assault at age 40 years. A table of estimates for the CIF at age 40 years is provided in the figure. The risk by 40 years is largest among the G2 for suicide (CIF=0.49%; 95% CIs=0.45% to 0.54%) and substance misuse mortality (CIF=0.35%; 95% CIs=0.30% to 0.41%). Both of these risks are larger than the respective risks by 40 years of age among the majority population (CIF for suicide=0.39% (95% CIs=0.37% to 0.42%); CIF for substance use=0.27% (95% CIs=0.24% to 0.32%)). Meanwhile, the risk by age 40 years for assault is 0.05% (95% CIs=0.04% to 0.06%) among the G2 and 0.02% (95% CIs=0.02% to 0.03%) in the majority population. For all other (known) causes of death, the G2 and majority population follow each other closely over age.

## Discussion

We have studied whether childhood socioeconomic disadvantage, measured by parental education, parental unemployment, parental disposable income and family situation, is associated with variation in young adult mortality rates for people born in Sweden to at least one parent born abroad (ie, the G2) versus the majority population of Sweden. We analysed mortality between 1992 and 2016 at ages 16–40 years. For the G2 at the generational level, the hazard rates of all-cause mortality, suicide, substance misuse, assault and unknown causes of-death were higher than the majority population in the minimally adjusted model. Hazard rates for the same causes of death remained higher in the fully adjusted model, although the HR was closer to one. In sensitivity analyses, we additionally showed that the higher hazard rate for assault mortality was only found among men and among G2 with two foreign-born parents. Even if the absolute risk of mortality from assault in Sweden is small, as the CIF in online supplemental figure S5 shows, this finding merits further attention. For the specific parental region of birth groups, those G2 with parent(s) born in Finland, other Nordic countries, Former Yugoslavia, the rest of Europe, sub-Saharan Africa, Northern Africa and Iran and Iraq had higher hazard rates of all-cause mortality and external mortality than the majority population in the minimally adjusted model. In the fully adjusted model, higher hazard rates of all-cause and external mortality only persisted for G2 with parent(s) born in Finland. Hazard rates of mortality from natural causes were comparable among the G2 versus the majority population.

Thus, we reveal that childhood SEB *is* associated with variation in the young adult mortality of the G2 relative to the majority population of Sweden. Indeed, for many of the specific parental countries/regions of birth groups, higher HRs in the minimally adjusted model are no longer elevated in the fully adjusted model. Although this is not the case at the generational level, the comparable lack of effect of childhood SEB on the HRs of mortality of all G2 combined is likely driven by the fact that G2 with parent(s) born in Finland as a group alone account for 31% of the total G2 risk time and 40% of all G2 deaths. Both all-cause and external-cause mortalities in this group remain highly elevated even after adjusting for childhood SEB.

Internationally, our findings contribute to a body of evidence that calls attention to the elevated mortality situation of young adult G2 in Europe.^4^ Our findings are consistent with studies that report higher all-cause mortality among the G2,^5^,^79 22^ particularly G2 who have parent(s) born in MENA countries and sub-Saharan Africa.^5^,^722^ In a literature that has focused largely on adult SEB and its association with G2 adult mortality,^5^,^9^ our main contribution is to demonstrate that this association exists for childhood SEB as well.

Strengths of this study included the use of total population register data that cover the entire resident population of Sweden. In this case, an examination of mortality among *all* those individuals who turned 16 years of age in Sweden between 1992 and 2016. The high quality of the registers means that very few individuals were excluded due to missing data on the key variables. The richness of the administrative register data, not least the ability to link information from parents to their children, permitted this analysis of the association between multiple measures of childhood socioeconomic disadvantage and young adult mortality, something that is not often possible due to a lack of available information. At the generational level, we were able to report differences between G2 and the majority population for a substantial number of causes of death. Furthermore, we were able to report differences between the G2 and majority population by detailed parent country/region of birth groupings, although for two much broader cause-of-death groups.

The first weakness of the study was its inability to look at detailed causes of death for the parental region of birth groupings. This was a consequence of mortality being a rare event at the ages we analysed, combined with the smaller population sizes and age structures of many parental origins. Second, between 3% (for the majority population) and 11% of deaths (among G2 with parent(s) born in Iran and Iraq) could not be classified because the cause of death could not be determined (ie, 797–799 codes for ICD-9 and R99 codes for ICD-10). Nevertheless, these unknown deaths *were* at least incorporated in the analyses of all-cause mortality and were also explicitly modelled at the generational level. Third, we reiterate that we implemented a delayed entry design, and as such, our results were conditional on survival until age 16 years old. Fourth, there are several shortcomings associated with HRs. These include misinterpretation of the HR as a relative risk, the use of a single estimate to cover large time scales (or ages) and selection bias due to variation in the underlying heterogeneity and frailty of the population at risk.^29 30^ Our provision of regression-standardised survival probabilities, failure probabilities, mortality rates and CIFs in the online supplementary materials helps to totally surmount many of these common issues.

Although our results are consistent with previous research, the ability to generalise them might be affected by factors unique to Sweden. First, the presence of a universal sociodemocratic welfare state in a country that also practices a migrant integration policy of inclusive multiculturalism as compared with countries with less expansive welfare models and/or integration policies (such as *assimilationist*). Second, there are differences in the migration history of Sweden, both in the countries that migrants were born in and moved from (eg, substantial flows from the other Nordic countries) or the reasons that people moved (eg, humanitarian). These important groups in Sweden may be smaller, or non-existent elsewhere. There may also be factors specific to being the child of, for example, a refugee which uniquely affect the all-cause and cause-specific mortality risks of the G2. Third, we examine a specific age range. 16–40 years old encompasses the mortality accident hump. This age range of mortality is dictated by death from external causes.^31^ Therefore, our findings are not generalisable to older G2 in Sweden or elsewhere that have reached ages where diseases and medical conditions are leading causes of death.

Overall, we have reported differences in the hazard rates of mortality from all causes and external causes of death at both the generational level and across a diverse range of parental regions of birth compared with the majority population of Sweden. This higher mortality is concentrated at ages in which the risk of mortality is very low (compared with infancy or older adult ages). Nevertheless, the higher mortality risk of the G2 still implies that this group is losing decades of potential life to causes of death that are entirely preventable. This higher mortality risk is associated with early life differences in parental education, parental income and parental unemployment compared with the majority population. This suggests that the childhood SEB of the G2 has a profound and lasting impact on their young adult mortality risks in Sweden. For current and future G2, social policies that are aimed at improving the socioeconomic situation of their migrant parents (and potential migrant parents) could ameliorate the mortality situation of their native-born children. The findings should also help to inform and improve public health policies, specifically national action programmes for suicide^32^ and substance misuse^33^ prevention. Such programmes emphasise the particular vulnerability of socioeconomically disadvantaged groups (of which there is overlap with the G2). However, they do not mention the G2, a subpopulation that could perhaps benefit from more nuanced and tailored intervention policies.

## supplementary material

10.1136/bmjph-2023-000643 online supplemental file 1

## Data Availability

Data may be obtained from a third party and are not publicly available.
