# Supplementary material for: Childhood socioeconomic background and elevated mortality among the young adult second generation in Sweden: a population-based cohort study
Source: BMJ Public Health. 2024 May 27;2(1):e000643. doi: 10.1136/bmjph-2023-000643 (PMC11812831; doi:10.1136/bmjph-2023-000643)
Supplement: online supplemental file 1 [file bmjph-2-1-s001.pdf]

Online supplementary materials

Table 1. Population sizes, time-at-risk (in years) ICD code categorisation, deaths, and mortality rates at the generational-level.

Cause-of-death	ICD-9 codes	ICD-10 codes	Majority population		Second-generation		
			N	Crude rate per 100,000	N	Crude rate per 100,000	Age-standardised rate per 100,000
**Population size**			1,930,551		415,282		
**Time-at-risk (years)**			24,827,172		4,814,834		
**Deaths**							
**All-cause**			10,783	43.4 (43.0-43.9)	2,621	54.4 (53.4-55.4)	54.4 (53.4-55.4)
**Natural causes**	*Inclusive of all codes below*	*Inclusive of all codes below*	3,315	13.4 (13.0-13.8)	625	13.0 (12.0-14.0)	13.3 (12.3-14.3)
Cancer	140-239	C00-D49	1,348	5.4 (5.1-5.7)	230	4.8 (2.4-5.4)	4.9 (2.5-5.5)
Circulatory	390-459	I00-I99	611	2.5 (2.3-2.7)	118	2.5 (2.1-2.9)	2.5 (2.1-2.9)
Respiratory	460-519	J00-J99	161	0.6 (0.6-0.8)	33	0.7 (0.5-1.0)	0.7 (0.5-1.0)
Endocrine, nutritional & metabolic	240-279	E00-E99	211	0.8 (0.7-1.0)	45	0.9 (0.7-1.3)	0.9 (0.7-1.3)
Nervous system	320-389	J00-J99	450	1.8 (1.7-2.0)	99	2.1 (1.7-2.5)	2.1 (1.7-2.5)
Other diseases & medical conditions	All other 000-799 codes NOT listed above	All other A-R codes NOT listed above	534	2.2 (2.0-2.3)	100	2.1 (1.7-2.5)	2.1 (1.7-2.5)
**External causes**	*Inclusive of all codes below*	*Inclusive of all codes below*	7,127	28.7 (28.3-29.1)	1,868	38.8 (37.7-40.7)	40.0 (39.0-41.0)
Suicides	950-959; 980-989	X60-X84; Y10-Y34	3,419	13.8 (13.3-14.2)	923	19.2 (17.8-20.1)	19.7 (18.3-20.6)
Substance misuse (inc. alcohol)	**Longer list of codes written in notes*	**Longer list of codes written in notes*	1,334	5.4 (5.1-5.7)	394	8.2 (7.4-9.0)	8.6 (7.8-9.4)
Traffic accidents	810-829	V00-V89	1,479	6.0 (5.7-6.3)	288	5.9 (5.3-6.7)	5.9 (5.3-6.7)
Assault	960-969	X92-Y09	210	0.8 (0.7-1.0)	112	2.3 (1.9-2.8)	2.3 (1.9-2.8)
Other external causes	All other 800-999 codes NOT listed above	All other V-Y codes NOT listed above	685	2.8 (2.6-3.0)	151	3.1 (2.7-3.7)	3.2 (2.8-3.8)
**Unknown causes of mortality**	797-799	R99	341	1.4 (1.2-1.5)	128	2.7 (2.2-3.2)	2.7 (2.2-3.2)

Notes: ICD9-codes for substance misuse (including alcohol): 291, 292, 303-305, 357.5, 425.5, 535.3, 571.0-571.3, 655.4, 760.71, 850-858; ICD-10 codes for substance misuse (including alcohol): F10-F16, F18-F19, G62.1, G31.2, G72.1, I42.6, K29.2, K70.0-K70.4, K70.9, K85.2, K86.0, Q86.0, PO4.3, & X40-X44.

Source: authors' calculations based upon register collection "Ageing Well".

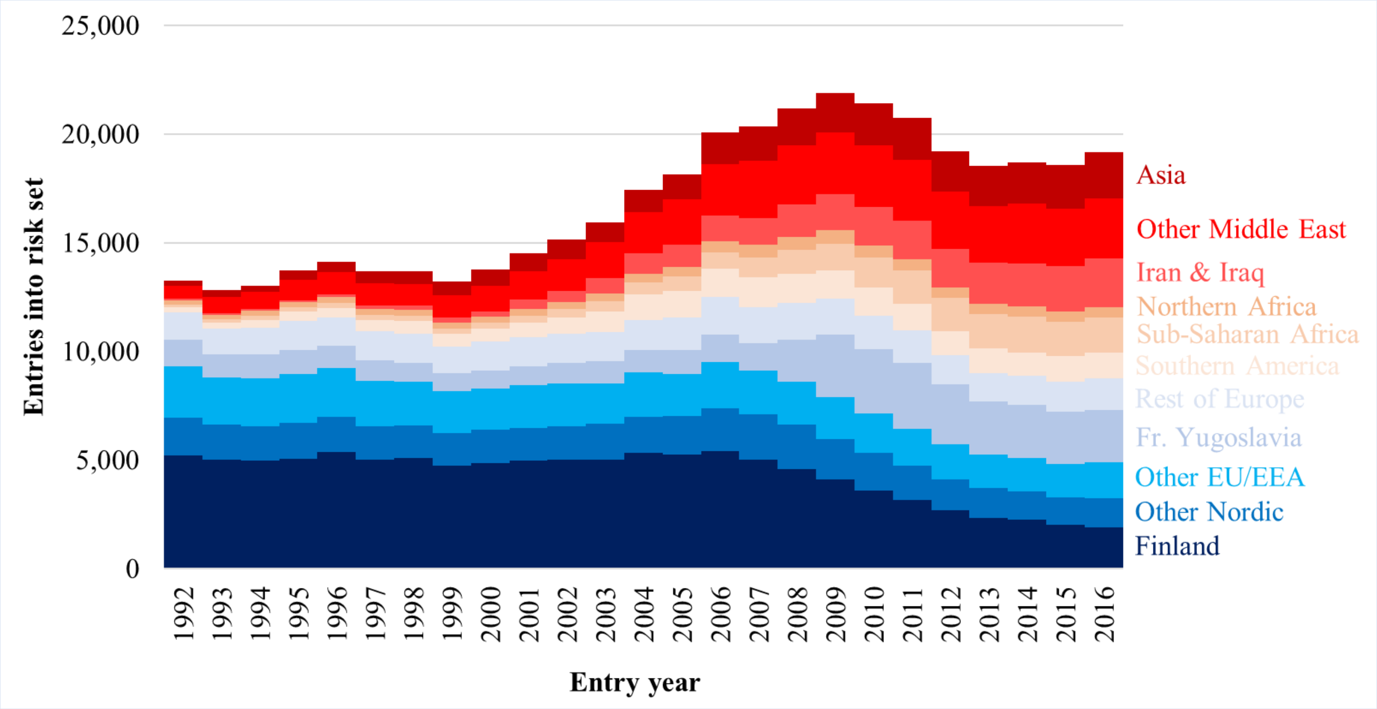

Figure 1.1. Number of annual second-generation entries to the risk set broken down by parental country/region of birth. *Source: authors' calculations based upon register collection "Ageing Well"*.

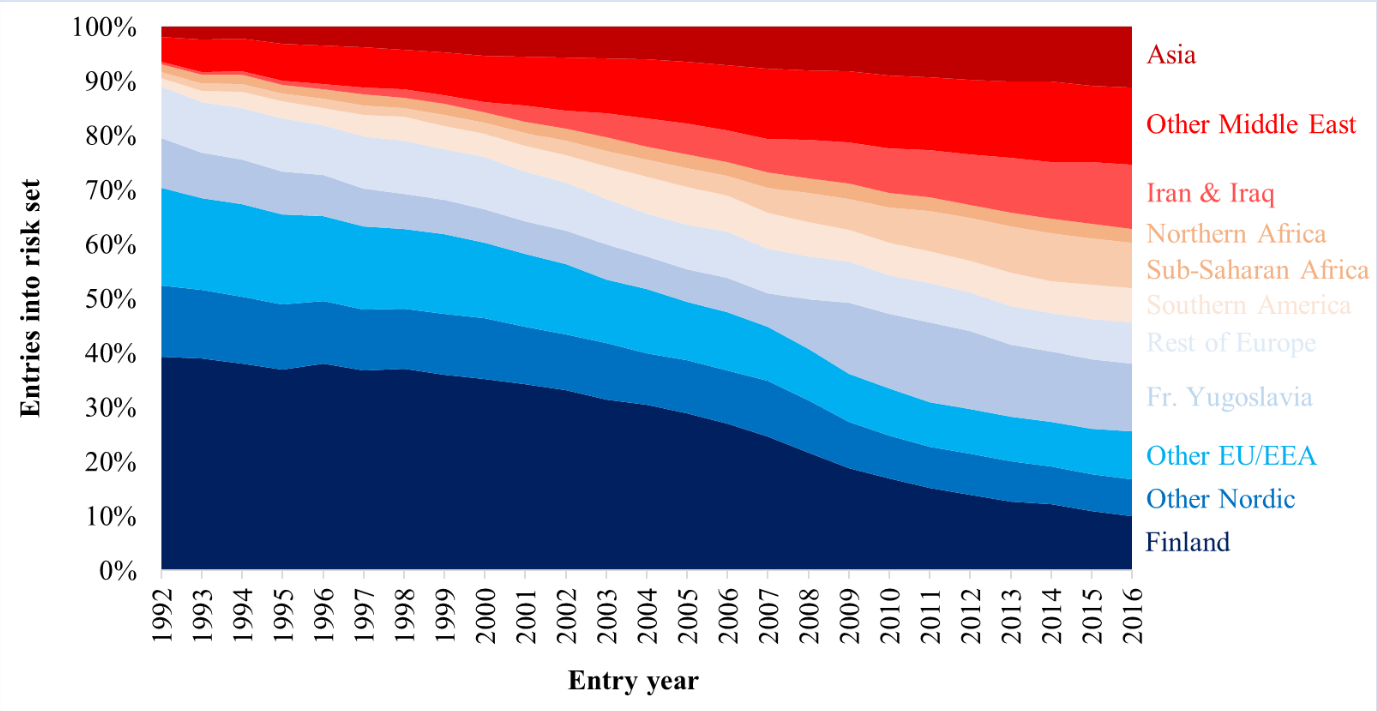

Figure 1.2. Percentage of annual second-generation entries to the risk set broken down by parental country/region of birth. *Source: authors' calculations based upon register collection "Ageing Well"*.

Table 2. Hazard ratios of the categories of the key predictor variables from the all-cause mortality model.

Predictor	HR	95%CIs
**Sex**		
Female		
Male	**2.38**	[2.29 - 2.47]
**Family & living situation**		
Living with married parents	Ref	
Living with cohabiting parents	**1.18**	[1.11 - 1.25]
Living with a single parent	**1.39**	[1.32 - 1.46]
Living alone	**2.43**	[2.09 - 2.82]
**Highest parental education level**		
Tertiary	Ref	
Secondary	**1.24**	[1.20 - 1.29]
Primary	**1.27**	[1.13 - 1.41]
**Average parental disposable income quintile**		
Highest	Ref	
High	1.02	[0.96 - 1.08]
Medium	1.02	[0.96 - 1.08]
Low	**1.16**	[1.09 - 1.24]
Lowest	**1.35**	[1.26 - 1.44]
**Average years of parental unemployment**		
0-years	Ref	
1-year	**1.17**	[1.12 - 1.22]
2-years	**1.23**	[1.14 - 1.32]
3-years	**1.65**	[1.38 - 1.94]

Notes: (1) Confidence intervals are specified at the 95% level (2) Estimates in bold have confidence intervals that do not overlap with 1. *Source: authors' calculations based upon register collection "Ageing Well"*.

Table 3. Hazard ratios of all-cause and cause-specific mortality at the generational-level, separate models for men and women.

**Second-generation status by cause-of-death**	**Men only**				**Women only**			
	Minimally Adjusted		Fully Adjusted		Minimally Adjusted		Fully Adjusted	
	HR	95%CIs	HR	95%CIs	HR	95%CIs	HR	95%CIs
Majority population	*Ref*		*Ref*		*Ref*		*Ref*	
All-cause mortality	**1.28**	[1.22 - 1.35]	**1.14**	[1.08 - 1.20]	**1.29**	[1.19 - 1.40]	**1.21**	[1.11 - 1.31]
Cancers	0.83	[0.68 - 1.01]	**0.74**	[0.60 - 0.90]	0.98	[0.81 - 1.20]	0.92	[0.75 - 1.12]
Circulatory	0.94	[0.74 - 1.19]	0.83	[0.66 - 1.06]	1.25	[0.88 - 1.77]	1.16	[0.82 - 1.65]
Respiratory	1.13	[0.72 - 1.75]	1.00	[0.64 - 1.56]	0.99	[0.49 - 2.02]	0.92	[0.45 - 1.88]
Endocrine, nutritional & metabolic	1.04	[0.67 - 1.59]	0.92	[0.60 - 1.41]	1.27	[0.78 - 2.07]	1.18	[0.73 - 1.93]
Nervous system	1.29	[0.99 - 1.67]	1.15	[0.88 - 1.49]	0.93	[0.63 - 1.40]	0.87	[0.58 - 1.30]
Other diseases & medical conditions	0.94	[0.69 - 1.28]	0.84	[0.62 - 1.13]	1.04	[0.77 - 1.41]	0.97	[0.72 - 1.31]
Suicides	**1.37**	[1.26 - 1.50]	**1.22**	[1.12 - 1.33]	**1.58**	[1.38 - 1.81]	**1.48**	[1.29 - 1.69]
Substance misuse (inc. alcohol)	**1.52**	[1.35 - 1.72]	**1.35**	[1.20 - 1.53]	**1.81**	[1.37 - 2.40]	**1.69**	[1.27 - 2.24]
Traffic accidents	1.05	[0.91 - 1.21]	0.93	[0.81 - 1.08]	0.97	[0.73 - 1.28]	0.90	[0.68 - 1.19]
Other accidents	1.11	[0.91 - 1.36]	0.99	[0.81 - 1.21]	1.37	[0.96 - 1.97]	1.28	[0.89 - 1.84]
Assault	**3.79**	[2.91 - 4.93]	**3.37**	[2.59 - 4.38]	1.16	[0.69 - 1.97]	1.08	[0.64 - 1.83]
Unknown causes of mortality	**1.89**	[1.48 - 2.40]	**1.68**	[1.32 - 2.13]	**2.28**	[1.56 - 3.33]	**2.12**	[1.45 - 3.11]

Notes: (1) Minimally-adjusted model includes birth year as a predictor (2) Fully-adjusted model additionally includes family situation, the highest level of parental education, parental disposable income, and parental unemployment (3) Confidence intervals are specified at the 95% level (4) Estimates in bold have confidence intervals that do not overlap with 1. *Source: authors' calculations based upon register collection "Ageing Well".*

Table 4. Hazard ratios of all-cause and cause-specific mortality among second-generation with two parents born abroad and second-generation with one parent born abroad.

**Second-generation status by cause-of-death**	**Both parents are foreign-born**				**One foreign-born, one Swedish-born parent**			
	Minimally adjusted		Fully-adjusted		Minimally adjusted		Fully-adjusted	
	HR	95%CIs	HR	95%CIs	HR	95%CIs	HR	95%CIs
Majority population	*Ref*		*Ref*		*Ref*		*Ref*	
All-cause mortality	**1.32**	[1.24 - 1.41]	**1.16**	[1.08 - 1.24]	**1.26**	[1.19 - 1.33]	**1.16**	[1.10 - 1.22]
Cancers	0.88	[0.71 - 1.11]	**0.78**	[0.62 - 0.97]	0.91	[0.76 - 1.08]	**0.83**	[0.70 - 0.99]
Circulatory	1.16	[0.86 - 1.55]	1.01	[0.76 - 1.36]	0.94	[0.73 - 1.21]	0.87	[0.67 - 1.11]
Respiratory	0.91	[0.48 - 1.73]	0.80	[0.42 - 1.52]	1.17	[0.75 - 1.83]	1.08	[0.69 - 1.68]
Endocrine, nutritional & metabolic	1.12	[0.67 - 1.85]	0.98	[0.59 - 1.63]	1.10	[0.74 - 1.64]	1.01	[0.68 - 1.51]
Nervous system	**1.44**	[1.06 - 1.96]	1.26	[0.93 - 1.72]	0.99	[0.75 - 1.32]	0.91	[0.68 - 1.22]
Other diseases & medical conditions	1.07	[0.78 - 1.49]	0.94	[0.68 - 1.31]	0.93	[0.71 - 1.22]	0.86	[0.65 - 1.12]
Suicides	**1.31**	[1.16 - 1.47]	**1.15**	[1.02 - 1.29]	**1.50**	[1.38 - 1.64]	**1.38**	[1.26 - 1.50]
Substance misuse (inc. alcohol)	**1.66**	[1.41 - 1.97]	**1.46**	[1.23 - 1.73]	**1.51**	[1.31 - 1.73]	**1.39**	[1.21 - 1.59]
Traffic accidents	0.94	[0.77 - 1.16]	0.83	[0.67 - 1.02]	1.07	[0.92 - 1.25]	0.98	[0.84 - 1.15]
Other accidents	1.22	[0.93 - 1.60]	1.07	[0.82 - 1.41]	1.14	[0.92 - 1.42]	1.05	[0.84 - 1.31]
Assault	**5.18**	[3.98 - 6.75]	**4.55**	[3.49 - 5.93]	1.23	[0.84 - 1.80]	1.13	[0.77 - 1.65]
Unknown causes of mortality	**2.16**	[1.60 - 2.90]	**1.89**	[1.41 - 2.55]	**1.89**	[1.47 - 2.43]	**1.74**	[1.35 - 2.23]

Notes: (1) Minimally-adjusted model includes birth year as a predictor (2) Fully-adjusted model additionally includes family situation, the highest level of parental education, parental disposable income, and parental unemployment (3) Confidence intervals are specified at the 95% level (4) Estimates in bold have confidence intervals that do not overlap with 1. *Source: authors' calculations based upon register collection "Ageing Well".*

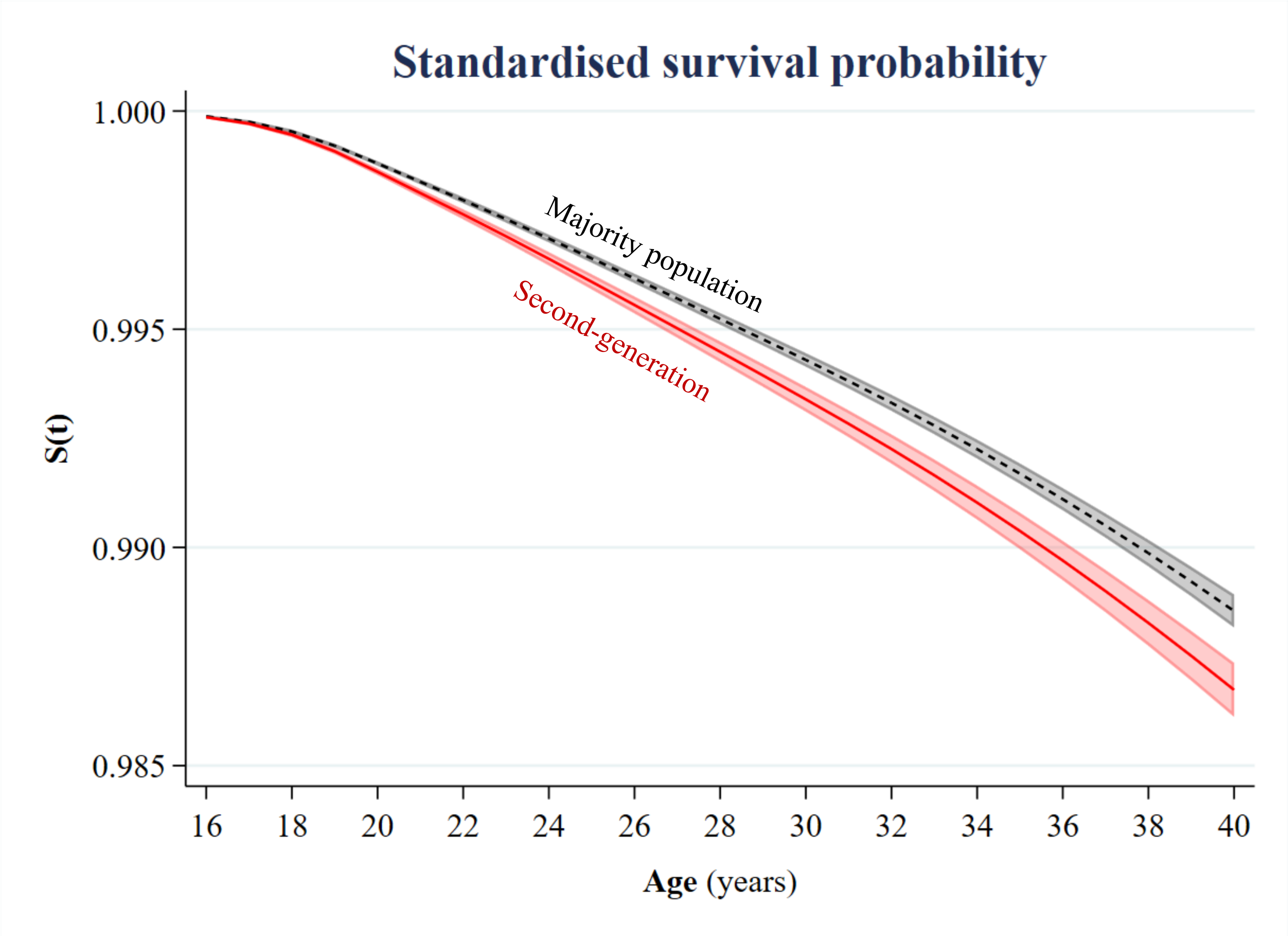

**Age**	**Standardised survival probability S(t)**					
(years)	Majority	95%CIs	G2	95%CIs	G2 — majority	95%CIs
16	0.99988	[0.99985 - 0.99991]	0.99986	[0.99983 - 0.99989]	-0.00002	[-0.00003 - -0.00001]
18	0.99953	[0.99949 - 0.99958]	0.99946	[0.99940 - 0.99951]	-0.00007	[-0.00010 - -0.00005]
20	0.99880	[0.99875 - 0.99885]	0.99861	[0.99853 - 0.99868]	-0.00019	[-0.00025 - -0.00013]
22	0.99796	[0.99789 - 0.99803]	0.99763	[0.99752 - 0.99775]	-0.00032	[-0.00042 - -0.00022]
24	0.99708	[0.99699 - 0.99717]	0.99661	[0.99646 - 0.99677]	-0.00046	[-0.00061 - -0.00032]
26	0.99616	[0.99606 - 0.99627]	0.99556	[0.99536 - 0.99575]	-0.00061	[-0.00080 - -0.00042]
28	0.99524	[0.99511 - 0.99537]	0.99448	[0.99425 - 0.99472]	-0.00075	[-0.00099 - -0.00052]
30	0.99430	[0.99414 - 0.99445]	0.99339	[0.99311 - 0.99368]	-0.00090	[-0.00118 - -0.00062]
32	0.99331	[0.99313 - 0.99350]	0.99226	[0.99192 - 0.99259]	-0.00106	[-0.00139 - -0.00073]
34	0.99225	[0.99204 - 0.99247]	0.99103	[0.99064 - 0.99142]	-0.00123	[-0.00160 - -0.00085]
36	0.99111	[0.99085 - 0.99136]	0.98970	[0.98925 - 0.99015]	-0.00141	[-0.00184 - -0.00097]
38	0.98987	[0.98957 - 0.99017]	0.98827	[0.98775 - 0.98879]	-0.00160	[-0.00209 - -0.00111]
40	0.98854	[0.98816 - 0.98892]	0.98674	[0.98612 - 0.98736]	-0.00181	[-0.00237 - -0.00125]

Figure 2. Regression-standardised (confounder-adjusted) survival probabilities among second-generation and majority population. Notes: (1) Estimated after fully-adjusted model, generational-level, all-cause mortality model (2) Confidence intervals specified at 95% level. (3) Table further includes "contrasts" between second-generation and majority population. *Source: authors' calculations based upon register collection "Ageing Well".*

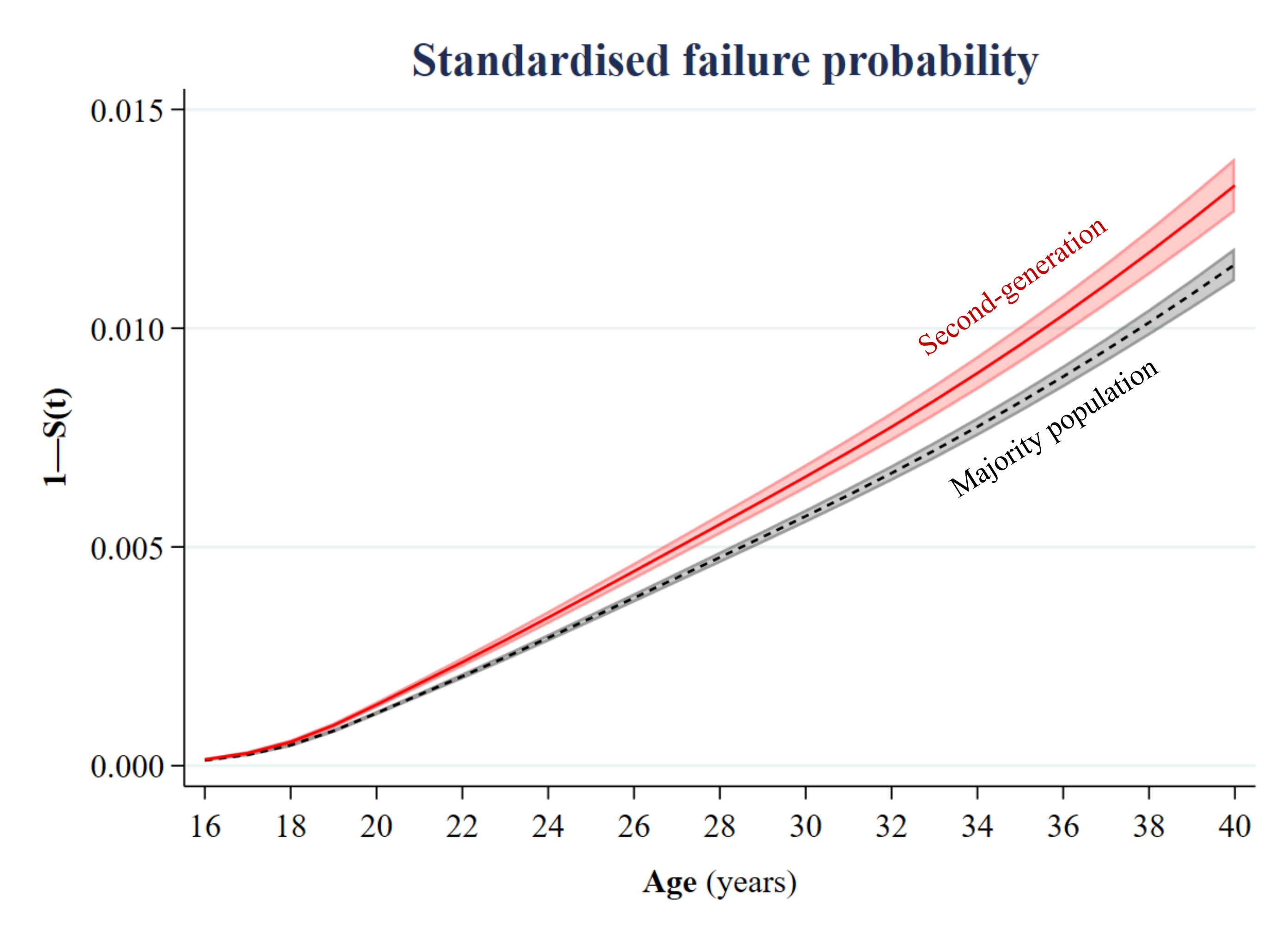

Age (years)	Standardised failure probability 1—S(t)					
	Majority	95%CIs	G2	95%CIs	G2 — majority	95%CIs
16	0.00012	[0.00010 - 0.00015]	0.00014	[0.00011 - 0.00018]	0.00002	[0.00001 - 0.00003]
18	0.00047	[0.00042 - 0.00052]	0.00054	[0.00049 - 0.00060]	0.00007	[0.00005 - 0.00010]
20	0.00120	[0.00115 - 0.00125]	0.00139	[0.00132 - 0.00147]	0.00019	[0.00013 - 0.00025]
22	0.00204	[0.00197 - 0.00211]	0.00237	[0.00226 - 0.00248]	0.00032	[0.00022 - 0.00042]
24	0.00292	[0.00283 - 0.00301]	0.00339	[0.00324 - 0.00354]	0.00046	[0.00032 - 0.00061]
26	0.00384	[0.00373 - 0.00395]	0.00444	[0.00426 - 0.00464]	0.00061	[0.00042 - 0.00080]
28	0.00476	[0.00463 - 0.00490]	0.00552	[0.00528 - 0.00576]	0.00075	[0.00052 - 0.00099]
30	0.00570	[0.00555 - 0.00586]	0.00661	[0.00633 - 0.00689]	0.00090	[0.00062 - 0.00118]
32	0.00669	[0.00650 - 0.00687]	0.00774	[0.00742 - 0.00808]	0.00106	[0.00073 - 0.00139]
34	0.00775	[0.00753 - 0.00797]	0.00897	[0.00859 - 0.00937]	0.00123	[0.00085 - 0.00160]
36	0.00889	[0.00864 - 0.00915]	0.01030	[0.00986 - 0.01076]	0.00141	[0.00097 - 0.00184]
38	0.01013	[0.00983 - 0.01044]	0.01173	[0.01122 - 0.01226]	0.00160	[0.00111 - 0.00209]
40	0.01146	[0.01108 - 0.01184]	0.01326	[0.01266 - 0.01390]	0.00181	[0.00125 - 0.00237]

Figure 3. Regression-standardised (confounder-adjusted) failure probabilities among second-generation and majority population. Notes: (1) Estimated after fully-adjusted model, generational-level, all-cause mortality model (2) Confidence intervals specified at 95% level. (3) Table further includes “contrasts” between second-generation and majority population. *Source: authors’ calculations based upon register collection “Ageing Well”.*

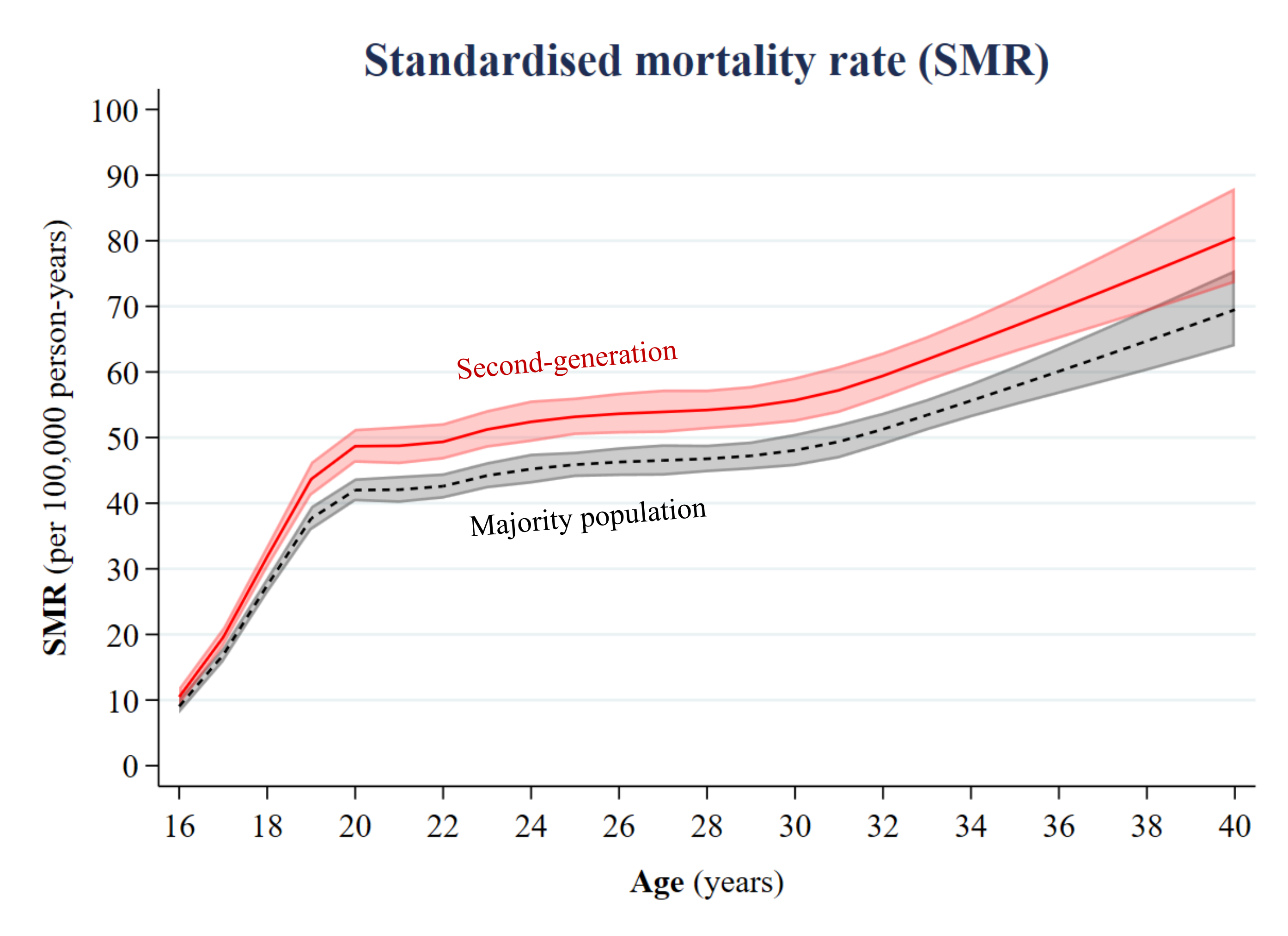

**Age** (years)	**SMR** per 100,000 PYs Majority	95%CIs	G2	95%CIs	G2 — majority	95%CIs
16	9.0	[8.0 - 10.3]	10.5	[9.2 - 11.9]	1.4	[1.2 - 1.7]
18	27.5	[26.2 - 28.7]	31.8	[30.1 - 33.7]	4.4	[3.8 - 4.9]
20	42.0	[40.3 - 43.8]	48.7	[46.2 - 51.3]	6.7	[5.9 - 7.6]
22	42.6	[40.7 - 44.6]	49.4	[46.7 - 52.2]	6.8	[6.0 - 7.6]
24	45.2	[43.0 - 47.6]	52.4	[49.3 - 55.7]	7.2	[6.3 - 8.1]
26	46.3	[44.1 - 48.5]	53.6	[50.6 - 56.8]	7.4	[6.5 - 8.3]
28	46.8	[44.7 - 48.9]	54.2	[51.2 - 57.3]	7.4	[6.5 - 8.4]
30	48.1	[45.6 - 50.6]	55.7	[52.4 - 59.2]	7.6	[6.7 - 8.6]
32	51.3	[48.9 - 53.8]	59.4	[56.0 - 63.0]	8.1	[7.2 - 9.2]
34	55.6	[53.1 - 58.3]	64.4	[60.8 - 68.3]	8.8	[7.8 - 10.0]
36	60.1	[56.6 - 63.7]	69.6	[65.1 - 74.5]	9.5	[8.4 - 10.8]
38	64.7	[60.2 - 69.6]	75.0	[69.2 - 81.2]	10.3	[9.0 - 11.6]
40	69.5	[63.9 - 75.5]	80.5	[73.6 - 88.1]	11.0	[9.7 - 12.5]

Figure 4. Regression-standardised (confounder-adjusted) mortality rates among the second-generation and majority population. Notes: (1) Estimated after fully-adjusted model, generational-level, all-cause mortality model (2) Confidence intervals specified at 95% level. (3) Table further includes "contrasts" between second-generation and majority population. *Source: authors' calculations based upon register collection "Ageing Well".*

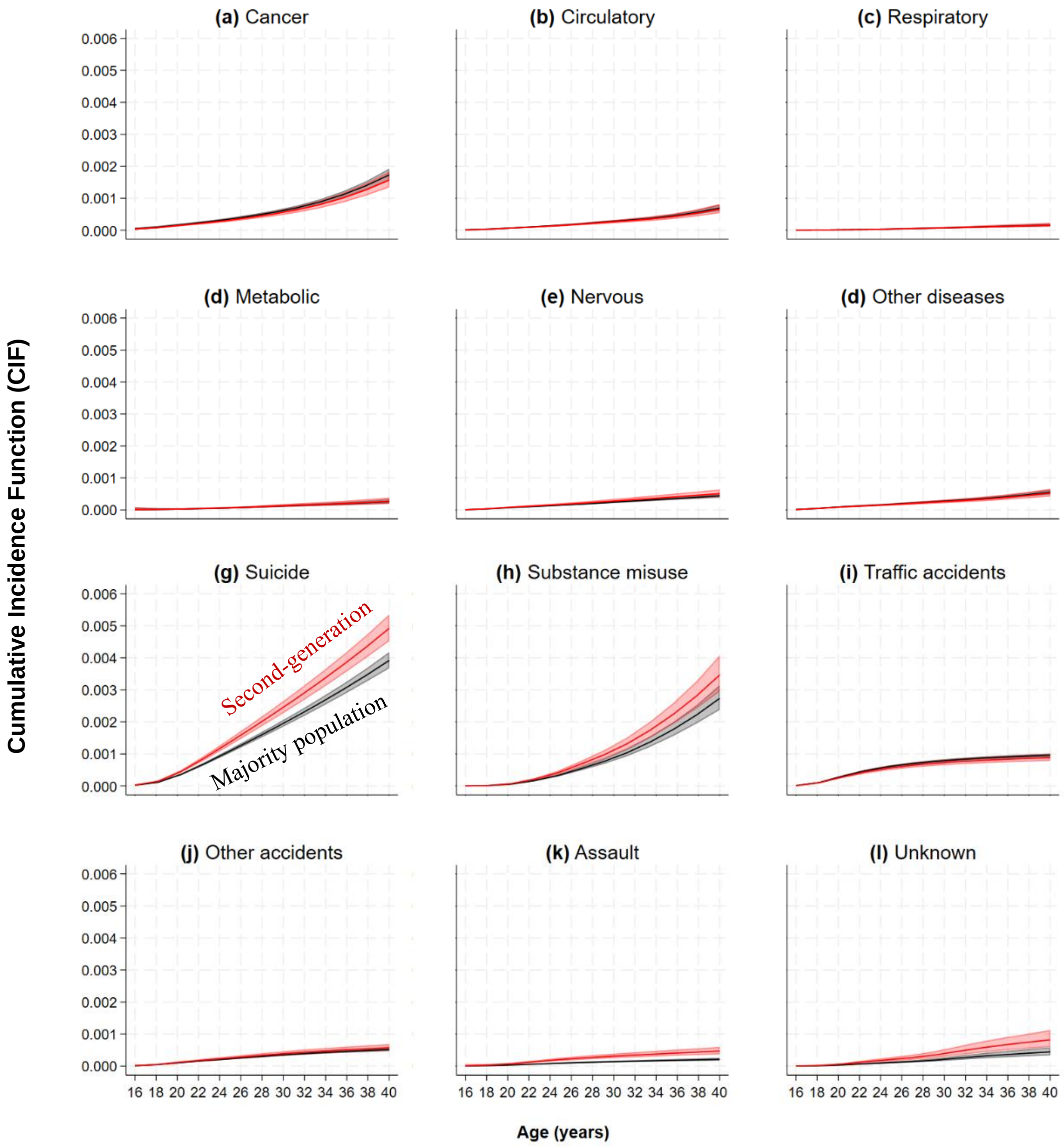

Cause-of-death	Majority population		Second-generation	
	CIF at age 40	95%CIs	CIF at age 40	95%CIs
Cancer	0.0017	[0.0016 - 0.0019]	0.0016	[0.0013 - 0.0018]
Circulatory	0.0007	[0.0006 - 0.0008]	0.0007	[0.0005 - 0.0008]
Respiratory	0.0002	[0.0001 - 0.0002]	0.0002	[0.0001 - 0.0002]
Endocrine, nutritional and metabolic	0.0003	[0.0002 - 0.0003]	0.0003	[0.0002 - 0.0004]
Nervous system	0.0004	[0.0004 - 0.0005]	0.0005	[0.0004 - 0.0006]
Other diseases and medical conditions	0.0006	[0.0005 - 0.0006]	0.0005	[0.0004 - 0.0007]
Suicide	0.0039	[0.0037 - 0.0042]	0.0049	[0.0045 - 0.0054]
Substance misuse	0.0027	[0.0024 - 0.0032]	0.0035	[0.0029 - 0.0041]
Traffic accidents	0.0010	[0.0009 - 0.0010]	0.0009	[0.0008 - 0.0010]
Other accidents and injuries	0.0005	[0.0005 - 0.0006]	0.0006	[0.0005 - 0.0007]
Assault	0.0002	[0.0002 - 0.0003]	0.0005	[0.0004 - 0.0006]
Unknown	0.0004	[0.0003 - 0.0006]	0.0008	[0.0006 - 0.0011]

Figure 5. Regression-standardised (confounder-adjusted) cumulative incidence function among the second-generation and majority population. Notes: (1) Estimated after fully-adjusted model, generational-level, cause-specific mortality model (2) Confidence intervals specified at 95% level. *Source: authors' calculations based upon register collection "Ageing Well".*
